# Patterns of Gene Expression in *Drosophila* InsP_3_ Receptor Mutant Larvae Reveal a Role for InsP_3_ Signaling in Carbohydrate and Energy Metabolism

**DOI:** 10.1371/journal.pone.0024105

**Published:** 2011-08-25

**Authors:** Satish Kumar, Debleena Dey, Gaiti Hasan

**Affiliations:** National Centre for Biological Sciences, Tata Institute of Fundamental Research, Bangalore, Karnataka, India; Yale School of Medicine, United States of America

## Abstract

**Background:**

The Inositol 1,4,5-trisphosphate receptor (InsP_3_R) is an InsP_3_ gated intracellular Ca^2+^-release channel. Characterization of *Drosophila* mutants for the InsP_3_R has demonstrated that InsP_3_-mediated Ca^2+^ release is required in *Drosophila* larvae for growth and viability.

**Methodology/Principal Findings:**

To understand the molecular basis of these growth defects a genome wide microarray analysis has been carried out with larval RNA obtained from a strong InsP_3_R mutant combination in which 1504 independent genes were differentially regulated with a log_2_ of fold change of 1 or more and *P*<0.05. This was followed by similar transcript analyses from InsP_3_R mutants where growth defects were either suppressed by introduction of a dominant suppressor or rescued by ectopic expression of an InsP_3_R transgene in the *Drosophila* insulin like peptide-2 (Dilp2) producing cells.

**Conclusions/Significance:**

These studies show that expression of transcripts related to carbohydrate and amine metabolism is altered in InsP_3_ receptor mutant larvae. Moreover, from a comparative analysis of genes that are regulated in the suppressed and rescued conditions with the mutant condition, it appears that the organism could use different combinations of pathways to restore a ‘normal’ growth state.

## Introduction

The efficient survival of multicellular organisms requires physiological co-ordination between cells, tissues and various organs. This co-ordination is achieved through signaling pathways some of which evolved in parallel with multicellular complexity. Release of calcium (Ca^2+^) from intracellular stores appears to be such a signaling pathway that co-evolved with metazoan life forms suggesting that it might be an important modulator of basic physiological pathways [1]. Our understanding of how intracellular Ca^2+^ signaling modulates systemic physiology however remains cursory. Intracellular Ca^2+^-release in response to inositol 1, 4, 5-trisphosphate (InsP_3_) signals occurs through a ligand-gated Ca^2+^ channel, the InsP_3_ receptor (InsP_3_R) present on the membranes of intracellular Ca^2+^ stores, primarily in the endoplasmic reticulum (ER). Vertebrate genomes have three genes for the InsP_3_R for which knock-outs have been generated in the mouse model [2], [3]. Among these, the InsP_3_R1 knock-out animals are lethal to a large extent. Analysis of the few survivors obtained showed growth defects and ataxia [3]. In the InsP_3_R2 and 3 knock-outs exocrine secretion is defective resulting in feeding defects [2].

The *Drosophila* genome contains a single gene for the InsP_3_R (*itpr*), making it an attractive genetic model for understanding systemic roles for InsP_3_-mediated Ca^2+^ release. There are several well characterized *itpr* mutant alleles of which the stronger alleles are larval lethal and exhibit severe growth defects prior to lethality [4], [5]. In order to understand the molecular basis of these growth defects we have carried out a series of transcriptional profiling experiments from *itpr* mutant animals in the absence and presence of either an extragenic suppressor or a rescuing transgene. The suppressor is the *Ca-P60A^Kum170ts^* mutant allele for the *Drosophila* sarco-endoplasmic reticular Ca^2+^-ATPase (SERCA), referred to as *Kum^170^* throughout this paper [6]. *Kum^170^* reduces the rate of Ca^2+^ uptake by the ER at 25°C in *Drosophila* neurons [7] and thus suppresses *itpr* mutant alleles by altering the dynamics of intracellular Ca^2+^ signals. The rescued condition has been described earlier and consists of expression of an *itpr^+^* cDNA transgene in insulin-producing cells (IPCs) of the brain [4]. In vertebrates changing levels of growth hormones and growth factors like the Insulin-like Growth Factors (IGFs) strongly affect body and organ size. In *Drosophila* there is no functional separation between insulin-like growth factors and insulin signaling. Thus a single insulin/IGF system manages growth and energy metabolism. The cellular and molecular basis of rescue of *itpr* mutants by expression of the *itpr^+^* cDNA in *Drosophila* IPCs remains to be understood.

Here, we characterized the transcriptional profile of *itpr* mutant larvae just prior to the manifestation of growth deficits, followed by a comparison of these changes in transcripts from suppressed and rescued mutant animals. Our data show that growth deficits are preceded by significant changes in gene expression and support a link between intracellular calcium signaling and energy metabolism. Reversal of the growth deficit either by suppression or rescue has helped identify candidate pathways and genes that might function downstream of intracellular Ca^2+^ release.

## Materials and Methods

### Drosophila melanogaster strains

*itpr^sv35/ug3^* is a larval lethal heteroallelic combination of single point mutants in the *itpr* gene generated in an ethyl methanesulfonate (EMS) screen. Detailed molecular information on these alleles has been published [5]. The embryonic wild-type *itpr* cDNA (*UASitpr^+^*) [8] was used for rescue experiments and *Ca-P60A^Kum170ts^* [6], [9] was used as a suppressor. The *dOrai^20119^* allele (referred to as *dOrai^2^*) was procured from the Bloomington Drosophila Stock Center [10]. *Dilp2GAL4* used for the rescue experiments was from Dr. E. Rulifson [11]. The wild-type Drosophila strain used in all experiments is *Canton-S* (*CS*). Fly strains were generated by standard genetic methods using individual mutant and transgenic fly lines described above. Flies were grown at 25°C in standard cornmeal medium containing agar, corn flour, sucrose, yeast extract along with anti-bacterial and anti-fungal agents.

### Larval staging

Staging experiments for obtaining molting profiles of heteroallelic mutant larvae were carried out as described previously with minor modifications [5]. Briefly, flies were allowed to lay eggs for a period of 8 hrs at 25°C. Embryos were allowed to hatch and grow further at this temperature. Larvae of the desired genotype were selected from these cultures at 56–64 h after egg laying (AEL) and transferred into vials of standard cornmeal medium lacking agar (agar less medium). Larvae were grown in agar less medium at 25°C and screened at the indicated time points for number of survivors and their phase of growth and development. For each time interval, a minimum of 75 larvae were screened in batches of 25 larvae each. Computation of means, SEM, and *t-*tests were performed using Origin 7.5 software (Origin Lab, Northampton, MA, USA).

### RNA isolation for microarray and qPCR

For isolation of total RNA, larvae of the requisite genotypes were selected at 58–62 hr AEL and snap frozen in liquid nitrogen. Total RNA was isolated using TRI Reagent (Sigma, St. Louis, USA) according to manufacturer's specifications. After quantification, RNA with a ratio of OD_260_/OD_280_>1.8 was taken for further experiments. Integrity of the isolated RNA was confirmed by the presence of full length rRNA bands on a 1.2% formaldehyde agarose gel. For microarrays three independent sets of RNA were isolated from control (*CS*), mutant (*itpr^sv35/ug3^*) and rescued larvae (*UASitpr^+^/+; Dilp2GAL4/+^/ug3^;* referred to as rescue) and two sets of RNA from *Kum^170ts^* suppressed larvae (*Kum^170ts^*/+; *itpr^sv35/ug3^;* referred to as suppressor). For qPCR three independent sets of RNA were isolated from all genotypes tested.

### Microarrays

Further quality control of isolated RNA, labeling of RNA probes with either cyanine 3-dUTP (Cy3) or cyanine 5-dUTP (Cy5), their hybridization to one Drosophila 8*60 K array AMADID: 27326 (Agilent Technologies Inc., Santa Clara, CA, USA) consisting of 8 replicate 60 K microarrays, scanning of signal intensities and analysis plus normalization of signal intensities were carried out by Genotypic Technology, Bangalore, India. Details of the numbers of probes present on the 60 K microarray are given in Table 1. Approximately 6000 EST probes could not be matched to existing CG IDs in FlyBase [10]. The experiments consisted of three arrays hybridized with control and mutant RNA (mutant), three arrays hybridized with mutant and *Dilp* rescue (rescue) and two arrays hybridized with mutant and *Kum* suppressor (suppressor) conditions. Independent sets of larvae were collected and used to isolate RNA for each of the hybridizations. The biological replicates were tested for reproducibility using the Pearson's correlation protocol from Microsoft Office Excel 2003. Correlation coefficients for biological replicates were>0.6 in all cases (Table 2). All data obtained is MIAME compliant and raw hybridization data from the microarray have been submitted to the Gene Expression Omnibus database (http://www.ncbi.nlm.nih.gov/geo) under series GSE29736.

**Table 1:** Details of probes spotted on the 8*60 K array, AMADID: 27326 from Agilent.

Number of Probes (minus control probes)	34169
Number of Probes with CG IDs	28023
Number of Unique CG IDs (Genes)	14157
Number of Probes without CG IDs (ESTs)	6146
Number of Unique Probes without CG IDs (ESTs)	5664

**Table 2:** Correlation analysis among the biological replicates of microarray hybridizations for RNA obtained from larvae of the genotypes *itpr^sv35/ug3^* (mutant), *UASitpr^+^; Dilp2GAL4/+; itpr^sv35/ug3^* (rescue) and *Kum^170/+^; itpr^sv35/ug3^* (suppressor).

	Mutant_1	Mutant_2	Mutant_3	Rescue_1	Rescue_2	Rescue_3	Suppressor_1	Suppressor_2
**Mutant_1**	1.00	0.86	0.79	0.06	0.04	0.06	0.03	0.04
**Mutant_2**		1.00	0.84	0.07	0.03	0.04	0.06	0.03
**Mutant_3**			1.00	0.11	0.10	0.01	0.15	0.14
**Rescue_1**				1.00	0.72	0.64	0.53	0.54
**Rescue_2**					1.00	0.83	0.53	0.66
**Rescue_3**						1.00	0.43	0.56
**Suppressor_1**							1.00	0.84
**Suppressor_2**								1.00

### Data analysis

Data analysis was performed using GeneSpring GX7.3.1 software (Silicon Genetics, Redwood City, CA, USA). Differentially regulated genes were ranked on the basis of signal intensity, normalized ratio, flag value and variance across biological replicate experiments. Top ranked genes had a higher intensity; high-normalized ratio for up and low for down; they were unflagged and showed very low variance or standard deviation. The normalized signal intensity of all filtered genes was taken for calculating log2 of the fold change in all experiments. The default multiple test correction in GeneSpring GX is the Benjamini and Hochberg False Discovery Rate protocol which provides a good balance between discovery of statistically significant genes and limitations of false positive occurrences. This, combined with a *t*-test performed at the 0.05% significant level for each gene, was used for calculating the final P-values. With this protocol the genes identified by chance (false discovery rate) is 5% of genes that are considered statistically significant.

Annotations of biological processes, molecular function and cellular localization were obtained using publicly available Gene Ontology information (The Gene Ontology Consortium) [12] uploaded to GeneSpring GX7.3.1 software and the publicly available DAVID database [13], [14] and FlyBase [10]. For comparison with published microarrays, lists of regulated genes with CG IDs were downloaded. Numbers of common genes were obtained by comparing the CG IDs from published data with our data using GeneSpring GX.

### Reverse Transcription-PCR (RT-PCR) and Real time PCR (qPCR)

Total RNA (1 µg) was reverse transcribed in a volume of 20 µl with 1 µl (200 U) Moloney murine leukemia virus (M-MLV) reverse transcriptase (Invitrogen Technologies, Carlsbad, CA, USA) using 1 µl (200 ng) random hexaprimers (MBI Fermentas, Glen Burnie, MD, USA) containing 1 mM dithiothreitol (DTT) (Invitrogen Technologies, Carlsbad, CA, USA), 2 mM of a dNTP mix (GE HealthCare, Buckinghamshire, UK) and 20 U of RNase Inhibitor (Promega, Madison, WI, USA) for 1 h at 42°C. The polymerase chain reactions (PCRs) were performed using 1 µl of cDNA as a template in a 25 µl reaction volume under appropriate conditions. Real-time quantitative PCR (qPCR) were performed on the Rotor-Gene 3000 (Corbett Research, Australia) operated with Rotor Gene software version 6.0.93 using MESA GREEN qPCR MasterMIX Plus for SYBR® Assay I dTTp (Eurogentec, Belgium). Experiments were performed with *rp49* primers as internal controls and primers specific to the gene of interest, using serial dilutions for the cDNA of 1∶10, 1∶100 and 1∶1000. Sequences of the primers used in the 5′ to 3′ direction are given below. Sequence of the forward primer is given first in each case.

*rp49* CGGATCGATATGCTAAGCTGT; GCGCTTGTTCGATCCGTA,

*CG2650* GCTCATCCTGCCCATTGTCA; TGGTTCAGCACCTTCAGCGT,

*unc-119* CGAGTTTCCTAACCTTCCACC; GGCGTAGTCGGCTTTGTTGT,

*CG9698* CCAGAGGTATCTTGGATGGAG; TCATTCAGACCACGGAAGTCC,

*rib* CGACACACCCATCCTGAGAA; TCGCCTCCACTTACTCCCAA,

*Cyp12d1-d* GATGGTATTCCGCAACGAGGG; ATCGTAGTTTTCCCCATGCCTC,

*DAT* CATGGCCCACACACTGGGT; GATCTTGGGAAACTCGTCGCTC,

*Idgf4* TCCTGCCCAATGTGAACTCTTCG; TCAGCTCGTAGATCGGTGCC,

*l(2)dtl* AAGGAGAAGGTGGACTGGCTGA; GGATTGGGAATGGGAGTGCGA,

*pan* TGCGTGCTAAGGTTGTTGCTG; CATTGGTATCTTGCTTCCGCTTC,

*Aldh* GAAACCATCAACCCGACCAC; ACATGCTGTAGGGCTTGCC,

*ng2* TCCTCGGCTGTGTGATGATCC; GTGAGGCTGTTGTTGTGGTGG.

Each qPCR experiment was repeated three times with independently isolated RNA samples. Cycling parameters were 95°C for 5 min, 40 cycles of 95°C for 15 s and 60°C for 30 s, 72°C for 30 s, then 1 cycle of 72°C for 5 min. The fluorescent signal produced from the amplicon was acquired at the end of each polymerization step at 72°C. A melt curve was performed after the assay to check for specificity of the reaction. The fold change of gene expression in the mutant relative to wild-type was determined by the comparative ΔΔC_t_ method [15]. In this method the fold change = 2^−ΔΔCt^ where ΔΔC_t_ =  (*C*_t(target gene)_ –*C*_t(rp49)_)_mutant_− (C_t(target gene)_ − C_t(rp49)_)_Wild type_.

## Results

### Growth defects in strong InsP_3_R mutants can be suppressed by reducing Ca^2+^ uptake into the endoplasmic reticulum Ca^2+^ store

**Figure 1:**
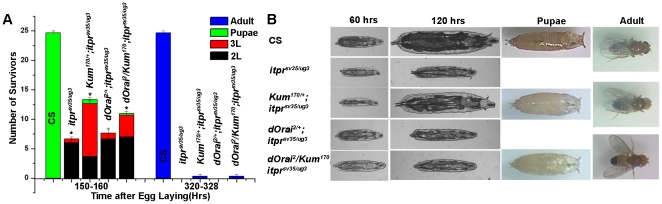
Lethality and growth defects in itpr^sv35/ug3^ mutant larvae can be partially suppressed by a single copy of *Ca-P60A^Kum170ts^*. (A) Number of surviving animals of the indicated genotypes and stages obtained at the specified times after egg laying. As compared to wild-type (*Canton-S or CS*), *itpr* mutant organisms (*itpr^sv35/ug3^*) are lethal. Two potential suppressor alleles, *Kum^170^* and *dOrai^2^*, were tested for suppression of lethality in *itpr^sv35/ug3^*. Results are expressed as mean survivors ± SEM and * indicates significance with *P*<0.01 (*t*-test). (B) Sample photographs of viable organisms of the indicated genotypes. *dOrai^2^* suppresses growth defects of *itpr^sv35/ug3^* to a greater extent at 60 hrs after egg laying as compared with *Kum^170^*. However, this effect does not persist and at 120 hrs hours after egg laying growth defects are suppressed more effectively by *Kum^170^*. A few adults with outspread wings, a phenotype reminiscent of viable *itpr* mutants, eclose from *itpr^sv35/ug3^* pupae in the presence of *Kum^170^*.

Strong hypomorphic alleles of the *Drosophila itpr* gene exhibit severe growth defects prior to lethality [4]. To understand the molecular changes and Ca^2+^ signals underlying these growth defects a dominant loss-of-function allele for the Sarco-endoplasmic reticulum Ca^2+^ ATPase (referred to as *Kum^170^*) was introduced in the lethal *itpr* heteroallelic mutant combination of *itpr^sv35/ug3^*. Previous studies have shown that the *Kum^170^* mutant allele can partially suppress phenotypes of weak *itpr* mutant alleles that arise during pupal development [9]. The cellular basis of this suppression is apparently elevated basal cytosolic Ca^2+^, which was measured in primary cultured neurons of the *Kum^170^/+* allele [7]. The elevation of basal Ca^2+^ observed is most likely due to a reduced rate of Ca^2+^ uptake from the cytosol to the ER store. In *itpr* mutant animals with one copy of the *Kum^170^* allele (*Kum^170^/+; itpr^sv35/ug3^*), a significant increase in larval viability was observed as compared with *itpr* mutants on their own (Figure 1A). The viable larvae grew to a size comparable to wild-type and a few pupated and eclosed as adults (Figure 1B). In contrast to *Kum^170^*, there was no significant suppression of growth and viability of *itpr^sv35/ug3^* animals by the allele *dOrai^2/+^* either on its own or in combination with *Kum^170/+^* (Figure 1A and B). The *dOrai^2/+^* allele is a gain-of-function allele for the *Drosophila* store-operated Ca^2+^ channel and suppresses *itpr* mutant phenotypes that arise during pupal development [7]. Since the *Kum^170^* allele was a more robust suppressor of growth and viability in *itpr* mutant larvae it was used in subsequent experiments.

### Transcriptional profiling of itpr^sv35/ug3^

**Figure 2:**
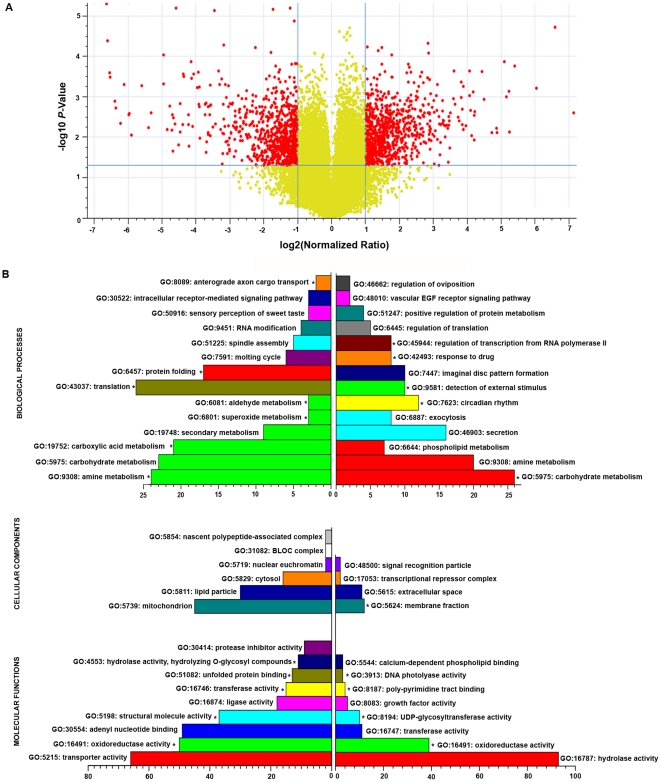
Microarray results of gene expression in *itpr^sv35/ug3^* larvae prior to cessation of growth. (A) A volcano plot of the expression level of all genes in *itpr^sv35/ug3^* as compared with wild-type. The X-axis defines the magnitude of the difference in expression between the mutant and the control state while the Y-axis defines the *P*-value of the observed change. Red dots represent genes whose expression was altered by one fold up or down upon taking log_2_ of the normalized expression ratios (X axis) with *P*<0.05 (−log_10_ of *P* value on the Y axis; −log_10_ of 0.05 = 1.3, horizontal blue line). (B) Gene Ontology (GO) classification of up-regulated and down-regulated genes in the listed categories. The X-axis represents the number of genes in the mutant in the marked category. Right panels indicate up-regulated genes and left panels indicate down-regulated genes. Only functional categories with P<0.05 are shown. * indicates functional categories with *P*<0.01.

To understand the molecular changes underlying growth defects in *itpr^sv35/ug3^* and their suppression a series of microarray experiments were performed. Initially, we obtained the transcriptional profile of RNA isolated from whole *itpr^sv35/ug3^* larvae, just before growth defects are evident (at 60 hrs after egg laying or AEL), and compared this with larvae of the wild-type *Canton-S* strain, in which genetic background the *itpr* mutants were originally generated. This experiment was repeated with three biological replicates and genes that were up and down-regulated (log_2_ fold change≥1; *P*<0.05) in *itpr^sv35/ug3^*, represented as red dots, are shown in the volcano plot in Figure 2A. Yellow dots represent genes that were not altered significantly by the above criteria. Amongst the 1504 independent genes that crossed this stringency threshold, 768 were up-regulated (right) and 736 were down-regulated (left). A complete list of these genes with individual hybridization intensity values and fold changes is given in the supplementary data (Table S1).

To identify specific pathways affected by reduced InsP_3_ receptor function genes with significantly altered transcription profiles were classified into established Gene Ontology (GO) categories. Groups of genes under a similar category whose numbers were significant (P<0.05) are depicted in the bar graphs in Figure 2B and C. Categories with higher significance (P<0.01) are marked with asterisks. In the mutant UP state 358 genes could be classified in various GO categories for biological processes (Table S2, BP), while in the mutant DOWN state 369 genes could be classified similarly (Table S2). From this analysis *itpr^sv35/ug3^* larvae show the greatest perturbation in genes affecting carbohydrate and amine metabolism. Genes affecting these two biological processes are both up-regulated and down-regulated and hence were analyzed further by placing them in metabolic pathways using the KEGG database. Down-regulated genes that classified under carbohydrate metabolism included Aconitase (TCA cycle), Enolase (Glycolysis), Zwischenferment (Pentose phosphate pathway) and several genes in galactose metabolism, indicating a reduction in the energy flux in *itpr* mutants. Up-regulated genes in carbohydrate metabolism were primarily in starch and sucrose pathways (e.g. amylase, maltase) and in amino sugar metabolism (chitinase). The chitinases overlap with genes up-regulated in amine metabolism. This group also includes enzymes that fall in pathways of tryptophan, arginine and proline metabolism. Amine metabolism genes that are down-regulated include several t-RNA synthetases and two enzymes affecting the one carbon pool by folate, another pathway that feeds into energy metabolism. Amongst other classes of up-regulated genes, of interest are a set affecting circadian rhythms, which include *clock, cycle, cry, dopamine transporter (DAT), serotonin receptor 1A* (*5HT1A*) and *norpA*. Some of these fall under other GO categories listed. For example *clock* and *cycle* are part of the list under positive regulation of transcriptional regulation, while *norpA* (encoding phospholipase Cβ) also comes under phospholipid metabolism. Genes encoding proteins regulating secretion and exocytosis such as *Synoptagmin 1, Synoptagmin 7, Synapsin*, and *Calcium activated protein for secretion* (*Caps*) are also up-regulated. In the set of genes significantly down-regulated under the translation category are several ribosomal proteins including mitochondrial ribosomal proteins. As expected from earlier findings genes belonging to the molting cycle are also down-regulated [16]. Next, genes differentially regulated in *itpr* mutant larvae were re-analyzed with GO terms for cellular compartments (CC) and molecular functions (MF) (Fig 2C, Table S2). The largest number of down-regulated genes localized to mitochondria supporting an early effect of reduced InsP_3_ receptor function on mitochondrial function and energy metabolism.

### Biological classification of differentially regulated genes in itpr mutants by transcriptional profiling after suppression of growth defects

**Figure 3:**
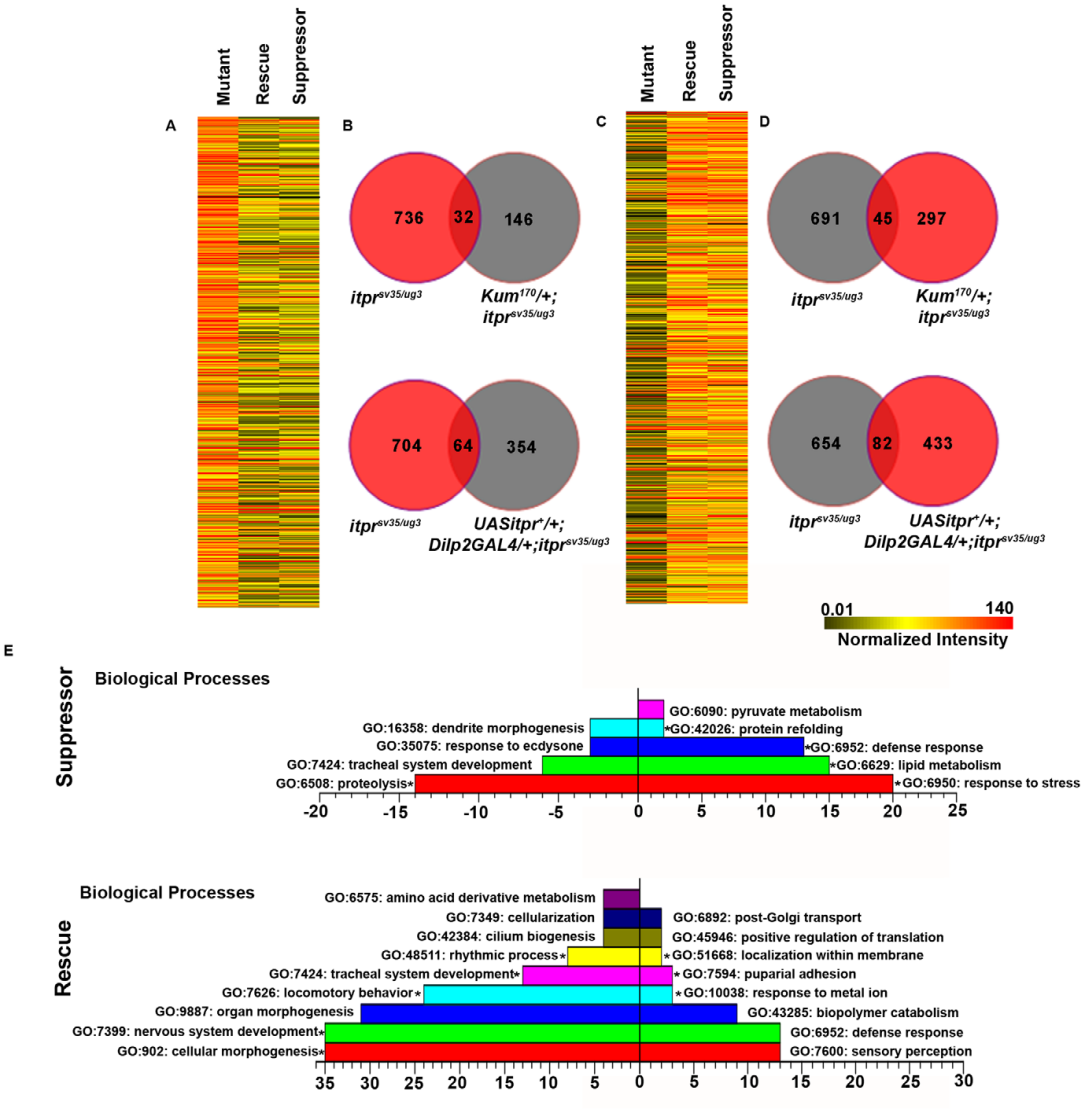
A comparison of gene expression changes in *itpr^sv35/ug3^* with suppressed and rescued conditions. The first column of the heat map in A shows the level of 768 up-regulated genes in mutant (log_2_ fold change ≥1 and *P*<0.05) and correspondingly their level in rescue (*UASitpr^+^/+; Dilp2GAL4/+; itpr^sv35/ug3^*) and suppressed conditions (*Kum^170^/+; itpr^sv35/ug3^*). Orange depicts up-regulation and black depicts down-regulation. The Venn diagrams (B) give the numbers of genes that overlap with up-regulated genes in the mutant and down-regulated in the rescue and suppressor individually. (C) Down-regulated genes (736) in the mutant (log_2_ fold change ≥1 and *P*<0.05) and their level of regulation in rescue and suppressed conditions with the corresponding Venn diagrams (D). Complete lists for up-regulated and down-regulated genes in mutants including one's that are rescued or suppressed are attached as supplementary information (Table S3, S4). E) GO classification of regulated genes in the suppressed and rescued condition (log2 fold change ≥1 and P<0.05) that are common with the mutant. The X-axis represents the number of genes in the suppressed and rescued condition in the marked category. Right panels indicate up-regulated genes and left panels indicate down-regulated genes. Biological processes with P<0.05 are shown. * indicates functional categories with *P*<0.01. Complete lists of these gene names are given in Tables S6 (suppressor) and S7 (rescue).

To identify pathways and genes likely to impact growth and viability most significantly, we measured whole genome transcript levels from RNA of *itpr* mutant larvae with a suppressor allele (*Kum^170^/+; itpr^sv35/ug3^*) and compared them to transcript levels in the *itpr* mutant (*itpr^sv35/ug3^;* Figure 3). To make this differential analysis more rigorous in parallel we carried out a comparison of RNA obtained from *itpr* mutant larvae rescued by expression of a *UASitpr* ^+^ transgene in *Drosophila* insulin-like peptide producing cells with *Dilp2GAL4.* These larvae exhibit normal growth similar to *Kum^170^/+; itpr^sv35/ug3^* animals [4]. Both rescue and suppressed conditions restored expression levels of a significant number of genes altered in *itpr^sv35/ug3^* (Figure 3; log2 fold change ≥1 and P<0.05). The heat maps show differential expression levels of genes in the *itpr* mutant when compared to wild-type (first column in Figure 3A and C). This was followed by comparison of expression levels in *itpr^sv35/ug3^* with either the *Dilp2GAL4* rescue (second column) or suppression by *Kum^170^* (third column) conditions. A complete list of these genes with individual hybridization intensity values and fold changes is available in the supplementary information for *Kum^170^* (Table S3) and for *Dilp2GAL4* rescue (Table S4). The numbers of genes whose expression levels returned towards the wild-type in the rescue or suppressed condition are depicted in the Venn diagrams (Fig 3B and D). A complete list of these genes with individual hybridization intensity values and fold changes is available in the supplementary information (Table S5).

To understand which biological processes are responsive to rescue/suppression transcriptionally altered genes in these conditions were classified into functional categories. As shown in Figure 3E the suppressed condition significantly (P<0.05) up-regulated expression level of genes for lipid and pyruvate metabolism, defense and stress responses. In the rescue condition different biological classes were up-regulated of which only defense response is common with the suppressed condition. Similarly for the down-regulated genes in suppressed and rescued conditions very few common processes were discovered. A similar analysis for GO categories that were suppressed and rescued both under cellular components and molecular functions also gave very few common components or functions (Figure S1). The complete lists of the genes in these GO categories are listed in Table S6 and S7. Taken together these observations suggest that suppression and rescue effect different processes to restore “normal” function.

Next we identified genes that were further up or down-regulated in the suppressed and rescued conditions. Among the up-regulated genes in mutants, 153 in the rescued condition and 85 in the suppressed condition were further up-regulated (Table S5). A total of 69 genes were up-regulated further in BOTH rescue and suppressed conditions (Table S5). The number of down-regulated genes in mutants that were further down-regulated in either suppressor or rescue was considerably fewer (Table S5). In this class there were 14 genes in rescue and 3 in the suppressor. None of these were common between the two conditions. A possible interpretation of this observation is that transcriptional up-regulation in *itpr* mutants is a compensatory mechanism which is further enhanced for some genes in the rescue and suppressed conditions. Genes that are down-regulated may be causative. Taken together these analyses suggest that rescued and suppressed conditions do not necessarily help the animal revert to its “normal” wild-type state.

The analyses so far helped to identify pathways that are differentially regulated in the various conditions tested. Next, we identified individual genes that may or may not cluster significantly in a GO pathway but whose expression is restored towards wild-type levels by a log_2_ fold change of 1 or more and P<0.05 in both rescue and suppressed conditions. Of the 15 genes identified in the UP mutant and DOWN rescue and suppressed states, 5 are predicted to effect oxidation-reduction processes and metabolism. There is one transcription factor (*ribbon*) known to effect development and up-regulated in larvae fed on a protein deficient diet of sugar only [17]; an alkaline phosphatase encoding gene (*CG8147*) which has earlier been identified as regulated by starvation and circadian rhythms [18], [19]; a circadian clock controlled gene *CG2650*, which is also regulated by altered mitochondrial function [20]. In the remaining genes, 5 were of unknown function, while the functions predicted for others are microtubule based movement (*CG10859*) and proteolysis with a possible role in immune function (*CG18180*; Table 3). *CG1659*, the *Drosophila* homolog of the *C.elegans* gene *unc119* has no known function but is highly expressed in the embryonic, larval and adult nervous system [10]. Among the genes of unknown function three (*CG18180*, *CG17974* and *CG12934)* are up-regulated when larvae are nutrient deprived [17]; in addition *CG12934* is up-regulated under starvation, while *CG18180* is up-regulated by altered mitochondrial function [20]. Genes that were down-regulated in the mutants and whose expression was restored in the rescue and suppressed condition are shown in Table 3. Of the 16 genes identified in the DOWN mutant and UP rescue and suppressed states, two (*CG32601* and *pangolin)* are transcriptional regulators of which *pangolin* is a repressor of *Wnt* signaling [21], *l(2)dtl* has homology with a ubiquitin ligase binding protein and is predicted to function in cell cycle regulation [22], [23] and insulin like peptide 5 is one of the *Drosophila* insulin peptides that function in metabolic control of energy utilization [24]. Two genes code for serine-type endopeptidases (*CG33459* and *CG12385*). Of these *CG33459* is up-regulated by JAK/STAT activation [25] and *CG12385* is down-regulated by starvation and stress [17], [26]. From the predicted functions of three other genes (*CG3752, CG13325, CG8525*) it is likely that these affect different aspects of metabolism. Previous microarray data from related conditions has shown that they are down-regulated as follows; *CG3725* under starvation [17], *CG13325* in larvae fed on sucrose only [17] and *CG8525* in an insulin signaling mutant (dFOXO; 26). Seven genes in this group are of unknown function. Among these *CG8317* is also down-regulated in sucrose fed larvae, though it is up-regulated under starvation [17].

**Table 3:** List of individual genes whose expression level is restored significantly in mutants by both rescue and suppression.

No.	CG_ID	Gene Name	Fold change			Biological Processes
			Mutant	Rescue	Suppressor	
1	*CG9698*	*-*	3.4	−1.6	−1.4	Oxidation reduction
2	*CG7461*	*-*	1.6	−1.7	−1.0	
3	*CG2065*	*-*	1.6	−2.5	−2.1	
4	*CG17843*	*-*	1.5	−1.2	−1.2	
5	*CG30489*	*Cyp12d1-p*	1.4	−1.8	−1.4	
6	*CG7230*	*rib*	2.9	−1.8	−1.6	Sequence-specific DNA binding transcription factor activity
7	*CG8147*	*-*	1.8	−1.3	−1.1	Metabolism, alkaline phosphatase activity
8	*CG2650*	*-*	4.9	−1.5	−1.9	Circadian rhythm
9	*CG10859*	*-*	2.0	−2.1	−1.3	Microtubule-based movement
10	*CG18180*	*-*	1.1	−1.2	−1.5	Serine-type endopeptidase activity,proteolysis
11	*CG1659*	*unc-119*	2.6	−1.3	−1.7	Unknown
12	*CG12880*	*-*	1.2	−1.0	−1.3	Unknown
13	*CG17974*	*-*	2.9	−1.6	−2.9	Unknown
14	*CG15905*	*-*	1.2	−2.0	−1.8	Unknown
15	*CG12934*	*-*	2.9	−2.3	−1.3	Unknown
16	*CG11295*	*l(2)dtl*	−4.7	4.4	3.6	Cell cycle regulation
17	*CG34403*	*pan*	−4.1	4.0	3.7	Transcriptional regulation
18	*CG32601*	*-*	−1.3	1.1	1.1	
19	*CG33273*	*Ilp5*	−3.0	1.9	2.5	Insulin receptor signaling pathway
20	*CG12385*	*θ Try*	−4.2	2.5	2.1	Serine-type endopeptidase activity
21	*CG33459*	*-*	−2.9	2.6	2.6	
22	*CG3752*	*Aldh*	−2.5	1.5	2.1	Aldehyde dehydrogenase (NAD) activity
23	*CG8525*	*-*	−1.6	1.9	1.8	Deoxyribose-phosphate aldolase activity
24	*CG13325*	*-*	−2.3	2.2	3.7	Transferase activity, transferring acyl groups other than amino-acyl groups
25	*CG8317*	*-*	−6.4	6.5	6.4	Unknown
26	*CG15741*	*-*	−4.7	2.4	5.3	Unknown
27	*CG12506*	*-*	−4.6	3.2	5.4	Unknown
28	*CG33268*	*-*	−3.9	3.9	4.2	Unknown
29	*CG7377*	*-*	−2.8	2.8	3.1	Unknown
30	*CG12491*	*-*	−1.7	2.4	2.6	Unknown
31	*CG10476*	*-*	−1.5	1.5	2.5	Unknown

The filtering for rescued and suppressed genes was the same as that for *itpr* mutants (log2 fold change ≥1 and P<0.05).

### Validation of selected target genes identified by the microarray screens and their normal tissue specific expression

**Figure 4:**
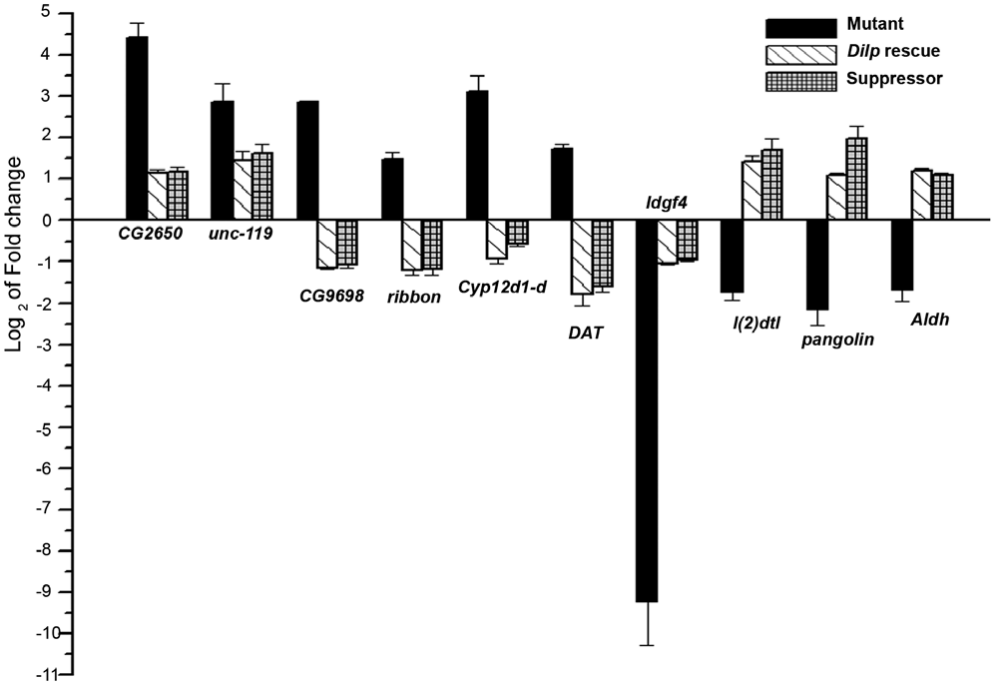
Validation of candidate genes by quantitative real time PCR (qPCR). The Y-axis represents the log_2_ of fold changes which were calculated by the ΔΔC_t_ method in which the C_t_ values of each gene were normalized to the level of a housekeeping gene (*rp49*) in control RNA from CS larvae. Each value is the mean ± SEM of three independent experiments, obtained from three independent RNA samples. The rescued and suppressed values were tested for significant difference from the mutants by Students t-tests followed by a Bonferroni correction for multiple tests. Except for *CG2650* and *unc119* all other genes were significantly altered (*P*<0.01).

**Figure 5:**
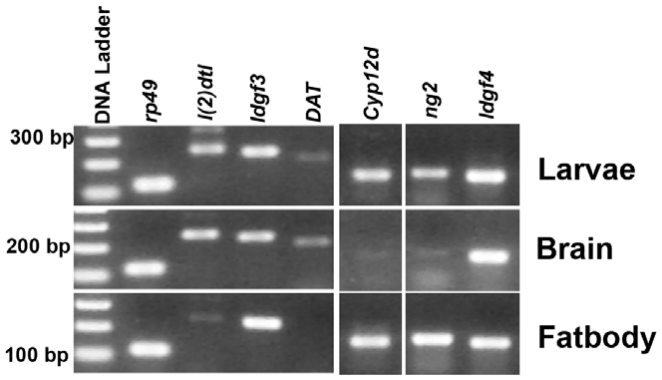
Expression of candidate genes in larval brains and fat bodies. Expression of candidate genes identified from the microarray analysis was tested in RNA from brains and fat bodies of third instar larvae by RT-PCR.

Quantitative real-time PCR (qPCR; Figure 4) was carried out to validate the altered expression level of selected genes that underwent significant changes in the mutant. In these experiments we tested candidates that were differentially regulated in rescued and suppressed conditions, as well as other genes that were of potential interest amongst the genes identified in the mutant condition only. Unlike the microarray where rescue and suppressed conditions were compared with mutant, here we quantified the expression levels of all conditions with RNA extracted from wild-type. Amongst the up-regulated genes we validated six genes in mutant, rescue and suppressor conditions, each of which was a candidate from a different biological process. In the rescue and suppressed conditions the expression level of two genes (*CG2650* and *CG1659*) was down-regulated when compared with the mutant, but remained high when compared with RNA from wild-type. The remaining four up-regulated genes validated in mutants were all down-regulated compared to wild-type. These included *Cyp12d1-d* (*CG33503*) and *DAT* (dopamine transporter with a neuronal function; *CG8380*; *Fumin*). In the microarray, *DAT* is regulated similarly but was filtered out due its higher P value. Amongst genes that were down-regulated in the mutant condition we tested *l(2)dtl* (*CG11295*), *Idgf4* (*CG1780*), *pangolin* (*CG34403*) and *CG3752*, all of which were validated as down-regulated in mutant and up-regulated in suppressor and rescue conditions by qPCR (Figure 4). Due to the filtering cut offs *Idgf4* does not appear in Table 3. To begin understanding the functional significance of these transcriptional changes the expression level of a subset of genes was also assessed in the brain and fat bodies of third instar larvae (Figure 5). Both these tissues have a central role in the control of energy metabolism in *Drosophila* and are known to express *itpr* transcripts at moderate levels [8], [10]. The genes tested were *l(2)dtl* and *DAT* which were found to be brain enriched, *cyp12d* and *ng2* which were enriched in the fat body and *Idgf3* and *Idgf4* which were expressed in both tissues. *ng2* and *Idgf3* were included since both are down-regulated significantly in mutants and up-regulated in the rescue condition. They were filtered out from the genes in Table 3 since they do not show a significant change in the suppressor.

## Discussion

Signaling through the InsP_3_ receptor and intracellular Ca^2+^ release are thought to affect multiple physiological conditions in mammals [27]. However, the underlying molecular mechanisms regulated or controlled by InsP_3_-mediated Ca^2+^ release in the context of specific physiological conditions still need elucidation. The genome-wide microarray analysis reported here with existing well-characterized InsP_3_R mutants has allowed us to investigate this question in the context of growth. From the gene ontology and pathway analyses of gene expression changes in the strong *itpr* mutant studied here, it is clear that metabolic genes particularly those related to carbohydrate and amine metabolism are significantly altered. The underlying cause(s) of these metabolic changes is of interest. One possibility, partially supported by our data as well as recent evidence from other groups [28] is a change in mitochondrial bioenergetics leading to subsequent effects on metabolic pathways. In the *itpr* mutant condition 50 down-regulated genes cluster in the mitochondrion (Fig 2; CC) and a highly significant number (P = 3.2E−5) have the molecular function (MF) of oxido-reduction. A set of genes classified as oxidoreductases are also up-regulated. However, these are primarily for detoxification and xenobiotic responses and are presumably up-regulated as a compensatory stress mechanism.

**Figure 6:**
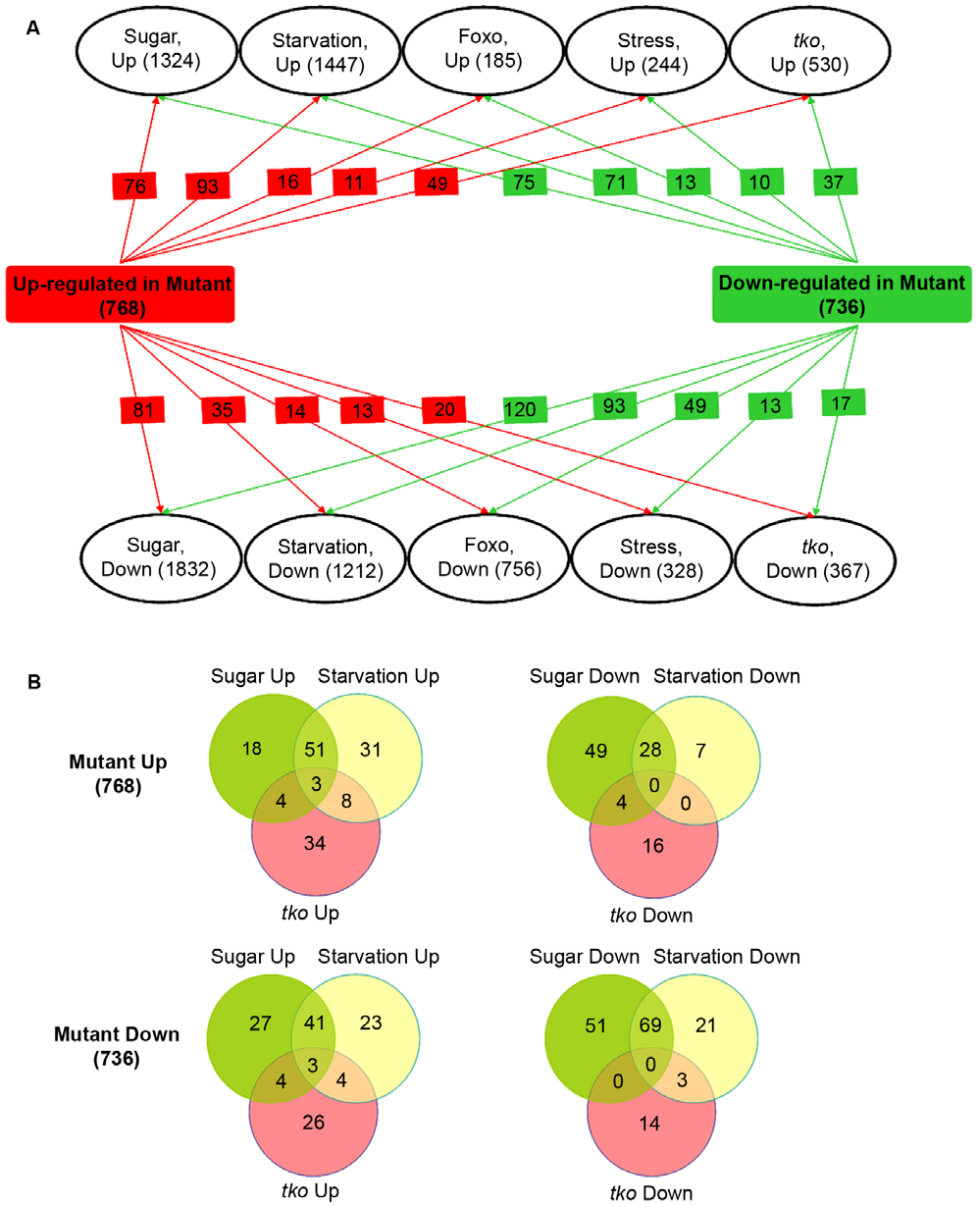
Comparative analysis of gene expression changes in *itpr^sv35/ug3^* with published data. (A) The list of genes significantly (*P*<0.05) up-regulated and down-regulated in mutant larvae were compared to gene lists obtained from selected published reports for larvae grown on sugar (protein deficient), under starvation, mutants for dFoxo and *tko*, and stress changes. Numbers of overlapping genes between these published conditions and those up-regulated and down-regulated in *itpr* mutants are listed in red and green square boxes. (B) Venn diagrams with the numbers of overlapping genes among the indicated conditions.

**Figure 7:**
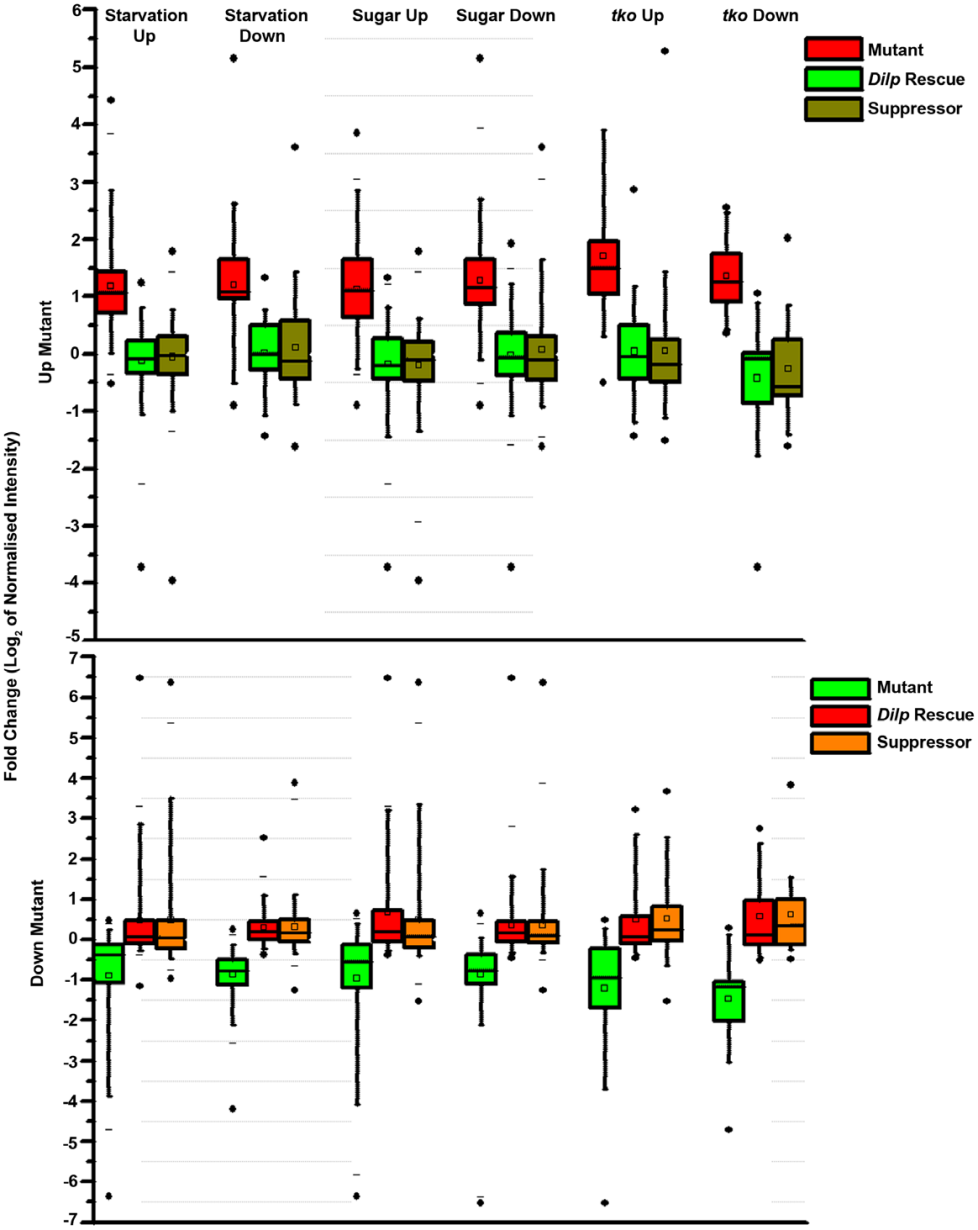
Expression levels of genes common between *itpr* mutants and the indicated conditions followed by their status in rescue and suppressed conditions. Box plots show the expression levels of genes common between *itpr* mutants and the indicated categories from figure 6. For genes up-regulated in mutants these include 93 genes for starvation up, 35 genes for starvation down, 76 genes for sugar up, 81 genes for sugar down, 49 genes for *tko* up and 20 genes for *tko* down. For genes down-regulated in mutants these include 71 genes in starvation up, 93 genes in starvation down, 75 genes in sugar up, 120 genes in sugar down, 17 genes in *tko* down and 37 genes in *tko* up.

Microarray studies in *Drosophila* with nutritionally altered, stress and mitochondrial mutant (*tko*) conditions have been published [17], [20], [26]. Therefore, we compared the list of up-regulated and down-regulated genes in our study with published gene lists for microarrays in what appear to be related conditions. In this analysis the maximum overlap obtained was between genes up-regulated in *itpr^sv35/ug3^* larvae with larvae undergoing starvation, followed by larvae grown on sugar alone (i.e. protein deficient), followed by the mitochondrial mutant *tko* (Figure 6A). The correlation with starvation conditions is not surprising since one of the earliest phenotypes of *itpr^sv35/ug3^* larvae is reduced feeding [4]. The change in circadian rhythm genes observed in the mutant condition may also be related to the feeding changes since circadian cycles are thought to integrate feeding and metabolic functions [29]. Even so the response of *itpr^sv35/ug3^* larvae does not correlate directly with either starvation or protein deficiency conditions or mitochondrial dysfunction, indicating that the transcriptional response to reduced intracellular Ca^2+^ release is complex (Figure 6B). Interestingly, the response of groups of genes that overlap with starvation, protein deficient and mitochondrial mutant conditions revert in the rescued and suppressed conditions (Figure 7). As suggested from Table S7, however, the extent of reversion differs in the two conditions, supporting the idea that the physiological states of rescued and suppressed larvae are different. In the rescued condition the *itpr* cDNA is expressed in a limited set of neuronal cells, while in the suppressed condition the dominant mutant for *Ca-P60A^Kum170ts^* affects the whole organism. Since the rescuing neurons are known to secrete several insulin-like peptides, which regulate cellular physiology through a pathway that acts via the transcription factor dFOXO, we also compared our data with microarray data from a *Foxo* mutant combination [30]. We do not observe a correlation with dFOXO regulated genes. Thus the transcriptional changes observed in *itpr^sv35/ug3^* must involve other gene regulatory factor(s) that are either directly responsive to changes in intracellular Ca^2+^ or the altered metabolic state of the organism. We analyzed our gene lists for possible candidates and identified *ribbon (CG7230), pangolin (CG34403)* and *l(2)dtl (CG11295)* as potential regulators of gene expression. None of these molecules appear to have a motif that would make them directly responsive to Ca^2+^ changes, suggesting that their regulation is indirect. The precise mechanisms of their regulation in *itpr* mutants need further study. Interestingly, *ribbon* is up-regulated in protein–deficient larvae, suggesting that in addition to its established role in regulation of development it could be a metabolic regulator [17]. No data are available at this stage to support a role for *pangolin* and *l(2)dtl* in the regulation of energy metabolism. Since *pangolin* is best known as a negative regulator of wingless signaling, we searched among the genes regulated in *itpr^sv35/ug3^* mutant for other components of the wingless pathway (Table S1). *wnt5* was up-regulated by a log2 fold change of 0.6 to 0.9 and marginally down-regulated in both suppressor and rescue conditions. In vertebrates, Wnt5 has been implicated in InsP_3_R activation during development [31]. *l(2)dtl* mutants in *Drosophila* are lethal as embryos and *l(2)dtl* transcripts are up-regulated under heat-shock [32]. The molecular *function* of *l(2)dtl* has not been investigated in *Drosophila.* In mouse the l(2)dtl homolog is known as CTD2. Several independent studies have shown that it plays an important role in cell proliferation through regulation of a G2/M checkpoint [22]. A functional analysis of these genes needs to be carried out in *Drosophila itpr* mutants. This will help validate their potential regulation by InsP_3_ mediated intracellular Ca^2+^ signaling and also in understanding how developmental pathways impact growth and metabolism.

## Supporting Information

Figure S1 **Gene Ontology classification for cellular components and molecular function (GO, CC and MF) of up-regulated and down-regulated genes in suppressed and rescued larvae.** The X-axis represents the number of genes in the suppressed and rescued condition in the marked category. Right panels indicate up-regulated genes and left panels indicate down-regulated genes. Number of genes in the categories shown had a P value≤0.05. * indicates functional categories with *P*<0.01. Complete lists of genes for each category are in Tables S6 and S7.
(TIF)

Table S1 **A list of significantly up-regulated and down-regulated genes (log_2_ fold change >1;** ***P*** **<0.05) in mutant larvae (** ***itpr^sv35/ug3^*** **).**
(XLS)

Table S2 **A list of significantly up-regulated and down-regulated GO categories (Biological Processes; BP, Cellular Component; CC, Molecular Functions; MF,** ***P*** **<0.05) with gene names in** ***itpr^sv35/ug3^*** **.**
(XLS)

Table S3 **A list of up-regulated and down-regulated genes with log_2_ fold change ≥1 and** ***P*** **<0.05 in suppressed larvae (** ***Kum^170/+^; itpr^sv35/ug3^*** **).**
(XLS)

Table S4 **A list of up-regulated and down-regulated genes with log_2_ fold change ≥1;** ***P*** **<0.05 in rescued larvae (** ***UASitpr^+^/+; Dilp2GAL4/+; itpr^sv35/ug3^*** **).**
(XLS)

Table S5 **A list of filtered (P<0.05) up-regulated and down-regulated genes that are common between mutant, rescued and suppressed larvae with fold changes and GO terms.**
(XLS)

Table S6 **A list of significantly up-regulated and down-regulated GO categories (BP, CC, MF,** ***P*** **<0.05) in suppressed larvae, with gene names.**
(XLS)

Table S7 **A list of significantly up-regulated and down-regulated GO categories (BP, CC, MF,** ***P*** **<0.05) in rescued larvae, with gene names.**
(XLS)

## References

[1] Cai X (2008). Unicellular Ca2+ signaling ‘toolkit’ at the origin of metazoa.. Mol Biol Evol.

[2] Futatsugi A, Nakamura T, Yamada MK, Ebisui E, Nakamura K (2005). IP3 receptor types 2 and 3 mediate exocrine secretion underlying energy metabolism.. Science.

[3] Matsumoto M, Nakagawa T, Inoue T, Nagata E, Tanaka K (1996). Ataxia and epileptic seizures in mice lacking type 1 inositol 1,4,5-trisphosphate receptor.. Nature.

[4] Agrawal N, Padmanabhan N, Hasan G (2009). Inositol 1,4,5- trisphosphate receptor function in Drosophila insulin producing cells.. PLoS One.

[5] Joshi R, Venkatesh K, Srinivas R, Nair S, Hasan G (2004). Genetic dissection of itpr gene function reveals a vital requirement in aminergic cells of Drosophila larvae.. Genetics.

[6] Sanyal S, Consoulas C, Kuromi H, Basole A, Mukai L (2005). Analysis of conditional paralytic mutants in Drosophila sarco-endoplasmic reticulum calcium ATPase reveals novel mechanisms for regulating membrane excitability.. Genetics.

[7] Venkiteswaran G, Hasan G (2009). Intracellular Ca2+ signaling and store-operated Ca2+ entry are required in Drosophila neurons for flight.. Proc Natl Acad Sci U S A.

[8] Venkatesh K, Siddhartha G, Joshi R, Patel S, Hasan G (2001). Interactions between the inositol 1,4,5-trisphosphate and cyclic AMP signaling pathways regulate larval molting in Drosophila.. Genetics.

[9] Banerjee S, Joshi R, Venkiteswaran G, Agrawal N, Srikanth S (2006). Compensation of inositol 1,4,5-trisphosphate receptor function by altering sarco-endoplasmic reticulum calcium ATPase activity in the Drosophila flight circuit.. J Neurosci.

[10] Tweedie S, Ashburner M, Falls K, Leyland P, McQuilton P (2009). FlyBase: enhancing Drosophila Gene Ontology annotations.. Nucleic Acids Res.

[11] Rulifson EJ, Kim SK, Nusse R (2002). Ablation of insulin-producing neurons in flies: growth and diabetic phenotypes.. Science.

[12] Ashburner M, Ball CA, Blake JA, Botstein D, Butler H (2000). Gene ontology: tool for the unification of biology. The Gene Ontology Consortium.. Nat Genet.

[13] Huang da W, Sherman BT, Lempicki RA (2009). Systematic and integrative analysis of large gene lists using DAVID bioinformatics resources.. Nat Protoc.

[14] Dennis G, Sherman BT, Hosack DA, Yang J, Gao W (2003). DAVID: Database for Annotation, Visualization, and Integrated Discovery.. Genome Biol.

[15] Lorentzos P, Kaiser T, Kennerson ML, Nicholson GA (2003). A rapid and definitive test for Charcot-Marie-Tooth 1A and hereditary neuropathy with liability to pressure palsies using multiplexed real-time PCR.. Genet Test.

[16] Venkatesh K, Hasan G (1997). Disruption of the IP3 receptor gene of Drosophila affects larval metamorphosis and ecdysone release.. Curr Biol.

[17] Zinke I, Schutz CS, Katzenberger JD, Bauer M, Pankratz MJ (2002). Nutrient control of gene expression in Drosophila: microarray analysis of starvation and sugar-dependent response.. EMBO J.

[18] Fujikawa K, Takahashi A, Nishimura A, Itoh M, Takano-Shimizu T (2009). Characteristics of genes up-regulated and down-regulated after 24 h starvation in the head of Drosophila.. Gene.

[19] McDonald MJ, Rosbash M (2001). Microarray analysis and organization of circadian gene expression in Drosophila.. Cell.

[20] Fernandez-Ayala DJ, Chen S, Kemppainen E, O'Dell KM, Jacobs HT (2010). Gene expression in a Drosophila model of mitochondrial disease.. PLoS One.

[21] Brunner E, Peter O, Schweizer L, Basler K (1997). pangolin encodes a Lef-1 homologue that acts downstream of Armadillo to transduce the Wingless signal in Drosophila.. Nature.

[22] Sansam CL, Shepard JL, Lai K, Ianari A, Danielian PS (2006). DTL/CDT2 is essential for both CDT1 regulation and the early G2/M checkpoint.. Genes Dev.

[23] Higa LA, Wu M, Ye T, Kobayashi R, Sun H (2006). CUL4-DDB1 ubiquitin ligase interacts with multiple WD40-repeat proteins and regulates histone methylation.. Nat Cell Biol.

[24] Gronke S, Clarke DF, Broughton S, Andrews TD, Partridge L (2010). Molecular evolution and functional characterization of Drosophila insulin-like peptides.. PLoS Genet.

[25] Bina S, Wright VM, Fisher KH, Milo M, Zeidler MP (2010). Transcriptional targets of Drosophila JAK/STAT pathway signalling as effectors of haematopoietic tumour formation.. EMBO Rep.

[26] Girardot F, Monnier V, Tricoire H (2004). Genome wide analysis of common and specific stress responses in adult drosophila melanogaster.. BMC Genomics.

[27] Berridge MJ, Bootman MD, Roderick HL (2003). Calcium signalling: dynamics, homeostasis and remodelling.. Nat Rev Mol Cell Biol.

[28] Cardenas C, Miller RA, Smith I, Bui T, Molgo J (2010). Essential regulation of cell bioenergetics by constitutive InsP3 receptor Ca2+ transfer to mitochondria.. Cell.

[29] Bass J, Takahashi JS (2010). Circadian integration of metabolism and energetics.. Science.

[30] Gershman B, Puig O, Hang L, Peitzsch RM, Tatar M (2007). High-resolution dynamics of the transcriptional response to nutrition in Drosophila: a key role for dFOXO.. Physiol Genomics.

[31] Kohn AD, Moon RT (2005). Wnt and calcium signaling: beta-catenin-independent pathways.. Cell Calcium.

[32] Kurzik-Dumke U, Neubauer M, Debes A (1996). Identification of a novel Drosophila melanogaster heat-shock gene, lethal(2)denticleless [l(2)dtl], coding for an 83-kDa protein.. Gene.

