# Supplementary figures and images for: Patterns of Gene Expression in Drosophila InsP3 Receptor Mutant Larvae Reveal a Role for InsP3 Signaling in Carbohydrate and Energy Metabolism
Source: PLoS One. 2011 Aug 25;6(8):e24105. doi: 10.1371/journal.pone.0024105 (PMC3162032; doi:10.1371/journal.pone.0024105)

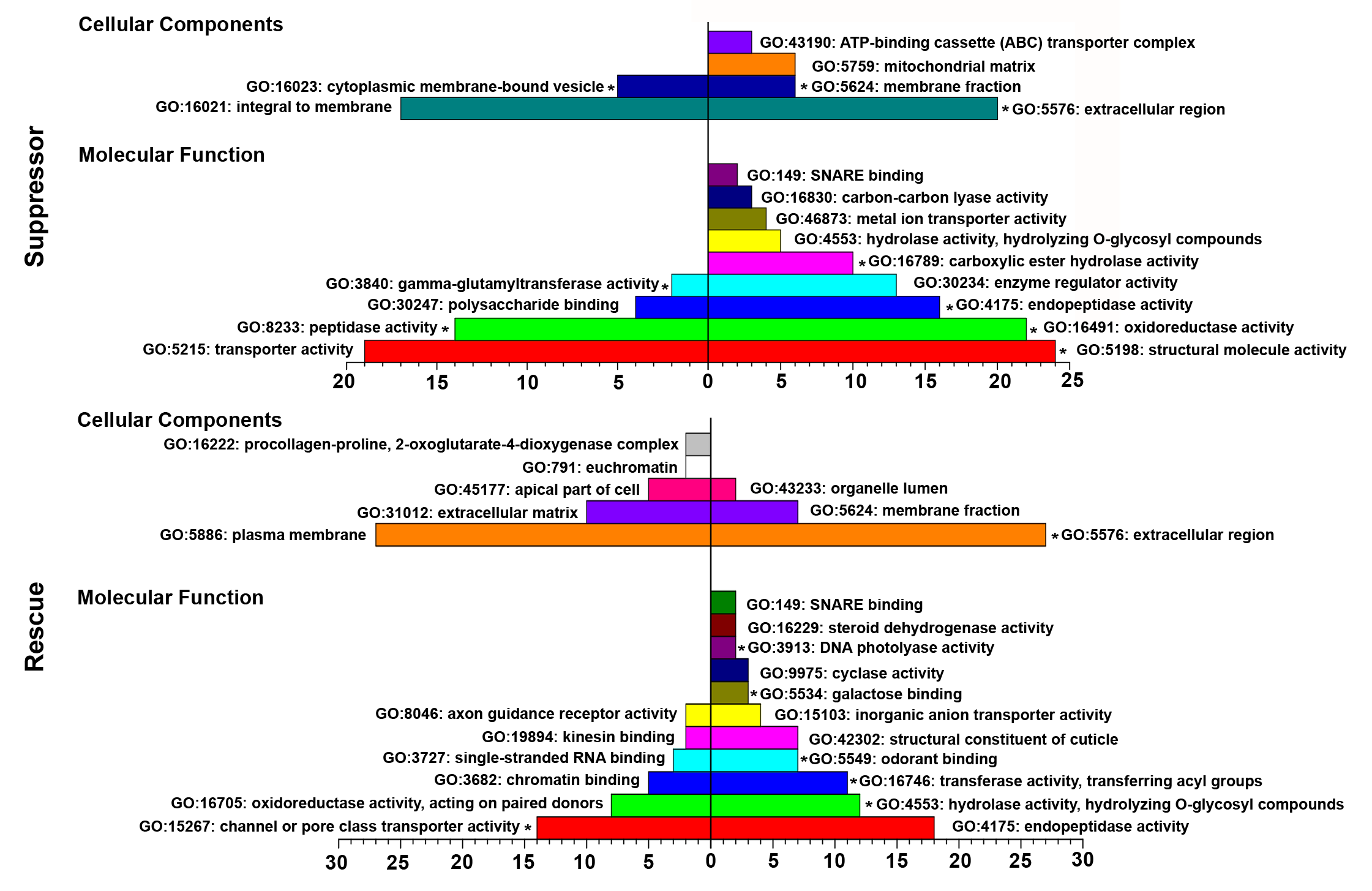

Supplement: Figure S1 — Gene Ontology classification for cellular components and molecular function (GO, CC and MF) of up-regulated and down-regulated genes in suppressed and rescued larvae. The X-axis represents the number of genes in the suppressed and rescued condition in the marked category. Right panels indicate up-regulated genes and left panels indicate down-regulated genes. Number of genes in the categories shown had a P value≤0.05. * indicates functional categories with P<0.01. Complete lists of genes for each category are in Tables S6 and S7. (TIF) [file pone.0024105.s001.tif]
